# Invasive Cane Toads: Social Facilitation Depends upon an Individual’s Personality

**DOI:** 10.1371/journal.pone.0102880

**Published:** 2014-07-17

**Authors:** Edna González-Bernal, Gregory P. Brown, Richard Shine

**Affiliations:** School of Biological Sciences A08, University of Sydney, Sydney, New South Wales, Australia; Helmholtz Centre for Environmental Research - UFZ, Germany

## Abstract

Individual variation in behavioural traits (including responses to social cues) may influence the success of invasive populations. We studied the relationship between sociality and personality in invasive cane toads (*Rhinella marina*) from a recently established population in tropical Australia. In our field experiments, we manipulated social cues (the presence of a feeding conspecific) near a food source. We captured and compared toads that only approached feeding sites where another toad was already present, with conspecifics that approached unoccupied feeding sites. Subsequent laboratory trials showed correlated personality differences (behavioural syndromes) between these two groups of toads. For example, toads that approached already-occupied rather than unoccupied feeding sites in the field, took longer to emerge from a shelter-site in standardized trials, suggesting these individuals are ‘shy’ (whereas toads that approached unoccupied feeding stations tended to be ‘bold’). Manipulating hunger levels did not abolish this difference. In feeding trials, a bold toad typically outcompeted a shy toad under conditions of low prey availability, but the outcome was reversed when multiple prey items were present. Thus, both personality types may be favored under different circumstances. This invasive population of toads contains individuals that exhibit a range of personalities, hinting at the existence of a wide range of social dynamics in taxa traditionally considered to be asocial.

## Introduction

Many scientific studies have attempted to predict ecological traits that render a species more or less likely to become a successful invader [1], [2], [3], [4]. However, behavioural traits such as ‘personality’ have been included only recently in this context [3], [4], [5], [6], [7], [8], [9]. Differences in behaviour among individuals of an invasive species might promote invasion success. For example, during the introduction and establishment stage, ‘bold’ behaviour may increase the chance of individuals finding their way into vehicles (cargo, etc.) to spread out from points of entry into new territory. Bold organisms also may be more prone to explore the new area, to utilize resources in highly disturbed sites, or to displace native competitors from preferred resources [6], [10], [11]. Once established, however, a group of organisms might be more able to succeed in colonizing new areas if they exhibit behavioural variation and/or a combination of behavioural types (i.e., individuals scattered along the bold-shy continuum) than if they are composed of only one type [5], [9], [12]. For example, range expansion in passerine birds (*Sialia mexicana*) is related to behavioural traits: more aggressive individuals displace interspecific competitors from places that eventually are occupied by less aggressive individuals [13].

The existence of behavioural varieties in a population also can promote or enhance social learning (learning that involves socially provided information) among individuals. One aspect of social learning that might play an important role during biological invasions is social facilitation, which occurs when the behaviour of an individual induces another individual to learn to exhibit the same behaviour [14] More generally, the presence of conspecifics provides evidence of habitat suitability and of resources. For example, an area that contains a feeding conspecific must contain food and competitors but is unlikely to contain predators [15], [16] We might thus expect organisms to use social information in different ways when making decisions about foraging sites and times. These differences in decision-making might also interact with personality – a ‘shy’ individual may be reluctant to approach a feeding site unless a conspecific is already there. For example, in guppies (*Poecilia reticulata*), shy female fish foraged in areas where they could detect another forager even when this was in conflict with their own knowledge (private information) about the best patch in which to feed; whereas bolder individuals avoided areas where a conspecific was present, probably in an attempt to reduce potential competition [17]. Social information; therefore, can be used differentially by individuals of different behavioural types (i.e., different behavioural syndromes). Behavioural syndromes are defined as a suite of two or more behaviours that are consistent within individuals over time or across ecological contexts, and that differ among individuals within a population [18], [19], [20].

We assessed sociality and its relationship with individual personality in an invasive species, the cane toad, *Rhinella marina,* in tropical Australia. Cane toads often use disturbed environments, and gather under artificial lights around buildings to feed on insects attracted by the lights [21]. This behaviour provides an opportunity for experimental manipulation of food resources (by providing lights), and aspects of the feeding situation – not only of abiotic cues such as substrate color and rugosity (that affect food availability [22]), but also social cues (the presence of conspecifics).

Like many invasive species, cane toads often attain high population densities in the years immediately following colonization of a new area [21], [23]. The high densities in recently-invaded areas (compared to the toads’ native range [24]) might enable novel social interactions. For example, under some conditions ‘bold’ toads (those that are willing to explore new resources) may be the most likely to be at the vanguard of an expanding population, whereas ‘shy’ individuals (that rely upon social cues from other toads before approaching novel stimuli) might benefit in established populations (from social cues given by bold individuals). The first step in evaluating this scenario is to see if a newly-established population does indeed contain individuals of varying behavioural proclivities. We explored this topic by conducting field experiments where we manipulated social cues (the presence of an already-feeding conspecific), and then ran laboratory trials to measure the personality of toads that approached the experimental units in the field. If the asocial individuals (those attracted to conspecific-absent trials) behaved differently during personality trials to social ones (those attracted to conspecific-present trials), the scenario would suggest the existence of a behavioural correlation between sociality and personality in this population of invasive cane toads. In turn, such behavioural variation might influence range expansion and colonization in this system.

## Materials and Methods

### Ethics Statement

The field experiments performed in this study were conducted at Beatrice Hill Farm, Northern Territory, Australia (12°38′S, 131°19′E). The site is operated as a research farm by the Northern Territory Department of Primary Industries who granted us access. The farm is not a National Park or other protected area. The study animal (*Rhinella marina*) is designated as a pest species and thus no permit from the relevant wildlife regulatory agency (Northern Territory Parks and Wildlife Commission) is required to study them. All procedures were approved by the University of Sydney Animal Care and Ethics Committee (Protocol #L04/4-2009/3/4999). Our study did not involve any endangered or protected species.

### Study Species and Area

Cane toads (*Rhinella marina*, or *Bufo marinus* in earlier literature) are large (to >1 kg) bufonid anurans. Native to tropical and subtropical areas of the Americas, the toads have been translocated to many countries in futile attempts to control insect pests of agriculture [25], [26]. Released in northeastern Australia in 1935, the toads have since spread across tropical Australia [27]. In 2005 the toad invasion front reached the Adelaide River floodplain 60 km east of the city of Darwin, in the wet-dry tropics of the Northern Territory (12°38′S, 131°19′E [28]). The current study is part of a broader ecological research program on cane toad biology, invasion, and impact [26], [29].

### Methods

We ran field experiments on free-ranging animals, to evaluate social facilitation and to objectively distinguish between ‘social’ and ‘asocial’ toads. We followed these field experiments by laboratory trials designed to identify differences in personality between these two groups of toads under controlled conditions, and experimentally tested the hypothesis that such behavioural differences might be simple effects of hunger level rather than underlying personalities of individual toads. We also used laboratory experiments to determine if differences in personality translate into differences in competitive foraging ability.

#### Field experiments

**Figure 1:**
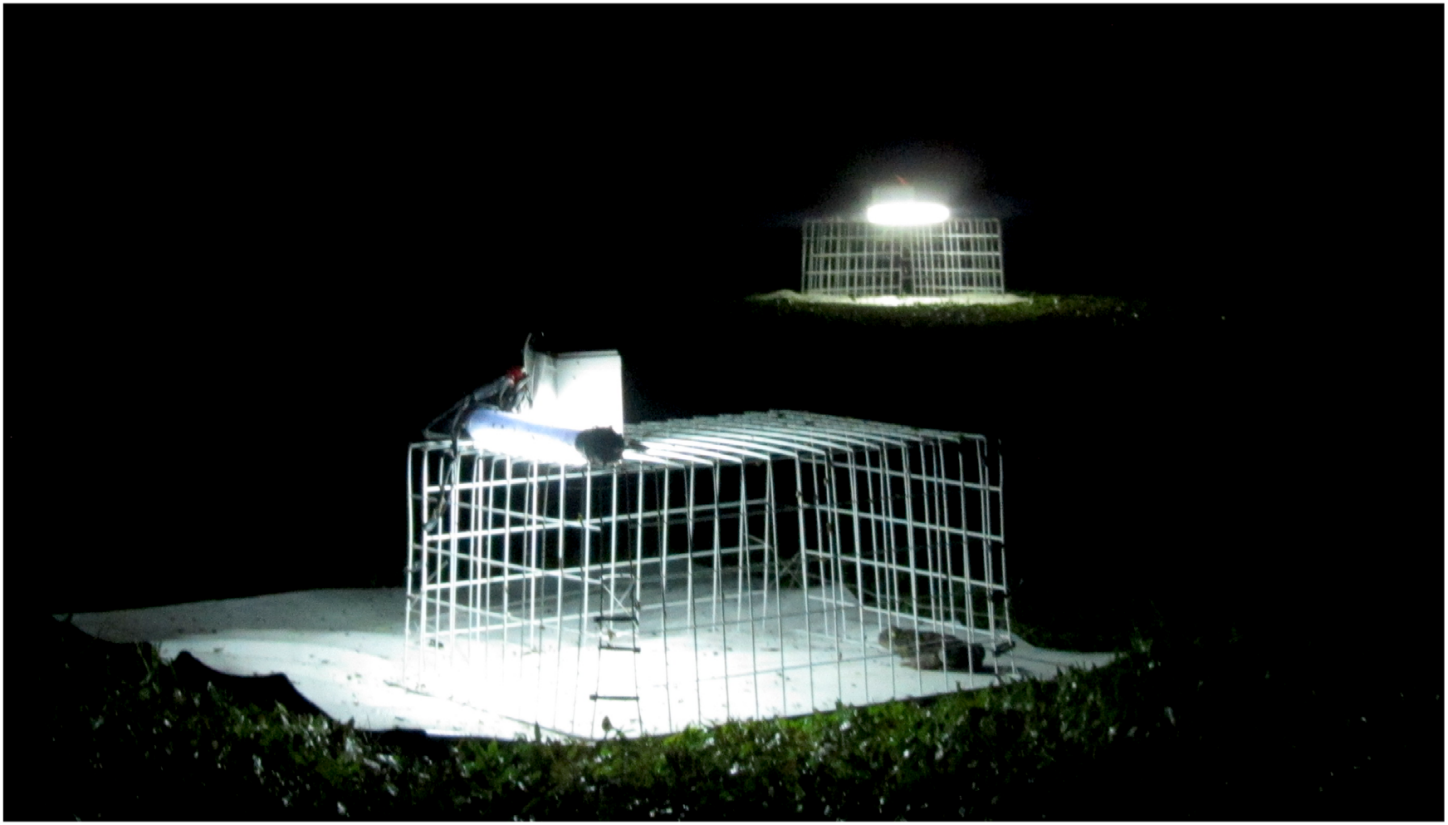
Experimental feeding-stations. Stations used to test the effects of conspecific presence on feeding responses of free-ranging cane toads. Note the captive toad in the foreground, providing a social stimulus.

To test whether the presence of a feeding toad facilitates recruitment of more toads to a feeding station, we modified a protocol previously used to assess foraging-site choice [22]. Four smooth white rubber mats (3.6-mm thick, 80×80 cm) were laid on the ground, 10 m apart, near known toad foraging sites. On top of each mat we placed a rectangular wire-mesh enclosure (50×40×24 cm) in which a toad could be detained. The white wire mesh formed 5×3 cm openings, with monofilament fishing line bisecting each opening to prevent toads from escaping. On top of each enclosure we placed a 250-mm fluorescent tube bulb (12 V, 8 W) to attract insects (Figure 1).

To provide a ‘social’ stimulus, we trained 32 adult female toads (98–120 mm snout-urostyle length [SUL]) to feed in the experimental apparatus (i.e., within a mesh enclosure on top of a white rubber mat, under a fluorescent light). Toads were induced to feed by placing crickets inside the enclosure with them until they became accustomed to the procedure. These training sessions (20–40 min long) were conducted for at least 10 days per individual prior to the field trials. The rest of the time, the trained toads were kept in outdoor containers (115×115×75 cm) with natural vegetation and water. Not all 32 toads were used every night, and we fed them every third night with medium-sized crickets.

To quantify recruitment of free-ranging toads to feeding stations, we set up the experimental units (at least 10 m apart) and turned on the lights after dusk. The enclosures of two of the units contained a pre-trained toad while the other two enclosures were empty. We observed the four experimental units from a distance of at least 5 m and collected the first free-ranging toad that approached the experimental unit. A total of 95 individuals approached our apparatus. For 44 toads we also measured individually “approach time”: the time elapsed from the moment that the light was turned on until the toad moved onto a mat and began feeding (which usually happened very soon after the toad moved onto the mat). Each trial ran from 2000–2200 h and the spatial arrangement of the treatments was randomly re-allocated every night.

Only the first toad that approached and began feeding on the mat was collected, individually marked with non-toxic paint and retained overnight in a clean, moist cloth bag. Toads that approached mats that had a feeding toad were classed as ‘social’, whereas those that approached empty mats were classed as ‘asocial’. The following morning, each toad was weighed and measured (SUL) and its sex was determined by morphology (skin color and granularity; presence of nuptial pads) and behaviour (release calls when held [30]). All individuals were then kept in outdoor containers (115×115×75 cm) with natural vegetation, food and water until tested in the following experiments. A subset of these toads was used in further (laboratory) trials on following nights (see below).

### Laboratory Experiments

#### Personality trials

We hypothesized that a toad’s sociality level (based on its willingness to approach an occupied versus unoccupied feeding station) depended upon its personality, with ‘shy’ toads requiring additional (social) cues whereas ‘bold’ individuals do not. If so, we would expect these toads to exhibit different responses to standardized situations in the laboratory. We ran trials to measure ‘boldness’ (time to exit a shelter and begin exploring a novel environment) in the toads collected during field experiments (above), under the assumption that bolder individuals will take less time to leave the shelter [31], [32], [33]. At 2000 h on the evening after the night of its capture, each toad was placed singly under a shelter in a large plastic container (115×115 cm floor area). The shelter was an inverted plastic bucket (27 cm in diameter, 25 cm high) with a 6×9 cm door facing the center of the enclosure. To allow each toad to settle down, the door was kept closed for the first 5 min of each trial. After this period we opened the door and video-recorded the toad’s activity over the next 60 min. Trials were run in near darkness, with a red light for illumination. From the video, we measured the time taken for the toad to fully exit the shelter. Only those toads that settled down during the 5 min acclimation period were tested (N = 63 out of 95 collected during the field experiments).

A toad’s willingness to leave a shelter sooner might be influenced by its hunger level rather than by some underlying behavioural dimension (boldness-shyness). To test this alternative interpretation, we repeated the experiment on 36 of the same toads on the following night. Half of the toads (from each personality type; bold toads n = 10, shy toads n = 8) were fed with crickets ad libitum 30 min before the start of the trial. To feed each toad, we placed it in a 60×36 cm enclosure with 10 medium size crickets. Most of the toads ate immediately, and we kept adding crickets until the toads stopped feeding. The other toads were not fed prior to their second trial (bold toads n = 10, shy toads n = 8).

#### Competition for food between ‘bold’ and ‘shy’ toads

We conducted two experiments to assess whether differences in personality (bold vs. shy) translated into differences in competitive foraging ability. We staged feeding competitions between pairs of toads, one shy and one bold (as categorized from the previous “personality” trials). In one experiment the toads competed over a single prey item and in the second experiment they competed over multiple prey items (see below). Prior to all feeding trials, toads were fasted for 48 h to standardize hunger level. Feeding trials were carried out between 2000–2230 h under dim illumination. The two experiments were run on different nights, with 55% of the toads tested in Experiment 1 also tested in Experiment 2.

#### Experiment 1

**Competition over a single prey item.** The toads were placed under shelters in opposing corners of a 60×36 cm enclosure to allow them to recover from manipulation. After 2 min, the shelters were lifted off both shelters at the same time and a single cricket was placed in the center of the enclosure. The trials began when the shelters were lifted and ended when the cricket was eaten (one toad wins one toad loses, so lack of independence is not an issue). We recorded the time elapsed before the cricket was eaten, which toad ate the cricket and the number of feeding attempts made by each toad. A total of 90 toads (45 pairs) were tested. In this experiment we used the 67 individuals tested on the personality trials plus 23 collected from our field trials. Competitors in each trial were size-matched.

#### Experiment 2

**Competition over multiple prey items.** For these trials we used larger enclosures (115×115 cm floor area), but followed the same methodology as for Experiment 1. Following 2 min acclimation of toads beneath shelters, 10 crickets were released at the center of the enclosure, and we recorded the number of crickets that each toad consumed. A total of 50 toads (25 pairs) out of the 67 tested for personality were used during this experiment.

### Statistical Analyses

#### Field experiment

We used ANOVA to assess whether the presence of a conspecific (social facilitation) affected the rate at which toads were recruited to feeding stations. The dependent variable was the time elapsed from the time we turned on the light of the feeding station until the first free-ranging toad entered and commenced feeding (‘approach time’). Only 39 of the 44 toads for which we recorded “time to approach” were included in the analysis, because five animals took longer than 90 min to approach. To quantify body condition (mass relative to length) of toads, we used residual scores from the general linear regression of ln body mass against ln SUL.

#### Laboratory experiments

We used ANOVA to compare the time taken to exit from a refuge (the dependent variable) between social and asocial toads (as categorized from field experiments i.e., asocial-bold vs. social-shy). For toads tested on two occasions, we calculated repeatability [34] to evaluate consistency within and between individuals in the time taken to exit a refuge. Only the toads that were tested in both trial 1 and trial 2 (N = 36) were included in the analysis. We used the scores of both trials to obtain a measure of personality, and included whether toads were fed or unfed as a factor in the model [34]. We ran this analysis using the statistical package R [35]. To assess whether a toad’s hunger level affected its time to leave a shelter, we used a repeated-measures analysis. For competition trials over a single prey item, we used contingency-table chi square tests to determine if personality (bold vs. shy) affected whether or not a toad succeeded in capturing the prey item (we excluded trials in which neither of the toads attempted to capture the prey). We used ANOVA to compare the time taken by bold versus shy toads to catch the prey, and the number of failed capture attempts. For feeding competition trials over multiple prey, we used ANOVA to compare the numbers of prey items consumed by bold versus shy toads. All analyses (except repeatability calculations) were run using JMP 9 (SAS Institute, Cary, NC) and we analyzed residuals to assess violation of ANOVA assumptions.

## Results

### Field Experiments

**Figure 2:**
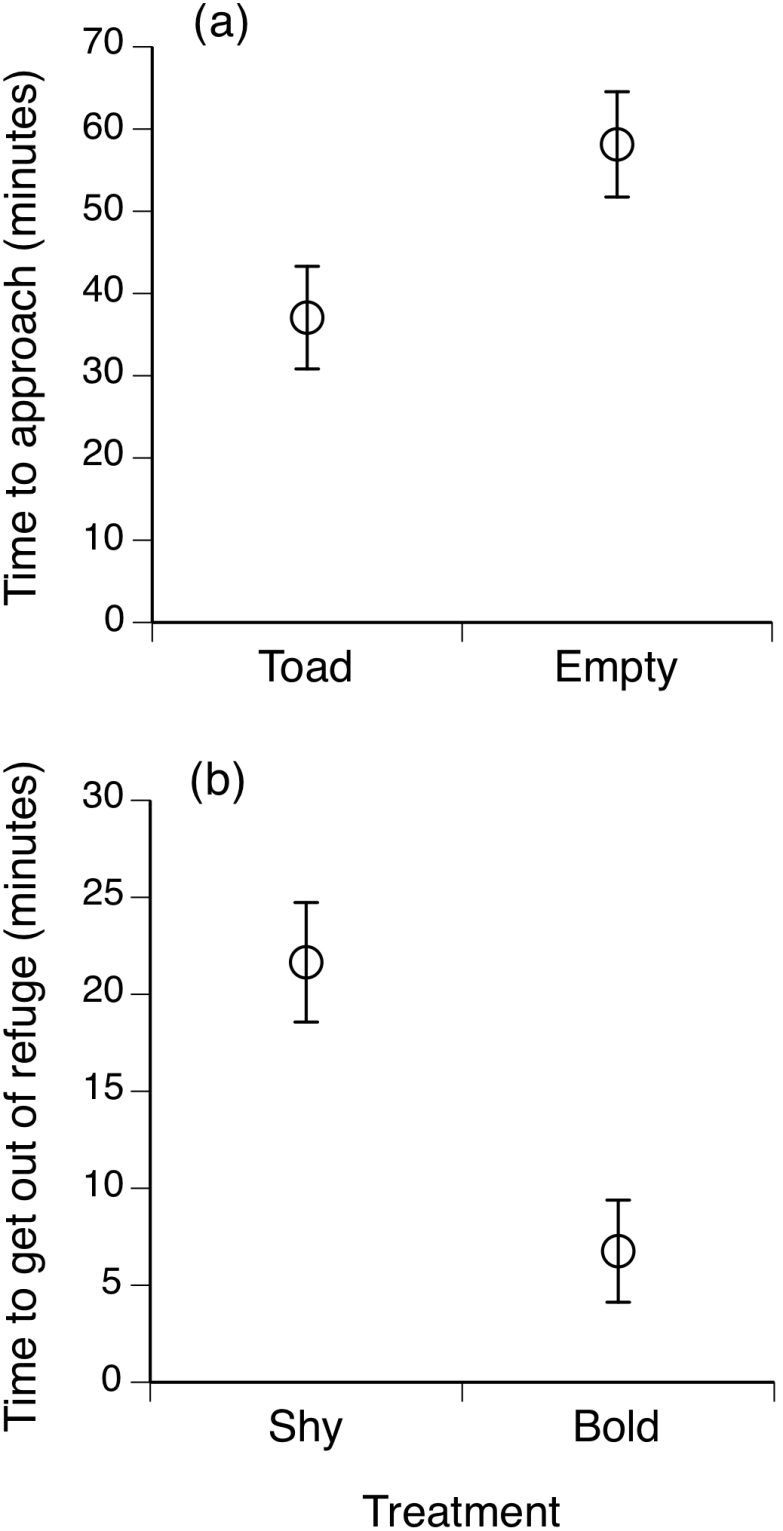
Behavioural divergence within cane toads. The upper panel shows the mean time taken for a free-ranging toad to approach an experimental feeding station and begin feeding, as a function of whether or not the station included an already-feeding conspecific toad. The lower panel shows the time taken for toads to exit from shelter items in laboratory trials. The two groups of toads compared in this panel are ‘bold’ and ‘shy’ animals, as categorized from their responses to experimental feeding stations in field trials conducted on the previous night. ‘Bold’ toads are those that approached an empty feeding station (asocial), whereas ‘shy’ toads are those that approached a feeding station containing a conspecific (social). The graphs show mean values and associated standard errors.

A total of 95 free-ranging cane toads approached our feeding stations: 23 females (11 social, 12 asocial), 40 juveniles (18 social, 22 asocial) and 32 males (16 social and 16 asocial). The time taken for a toad to approach the experimental light was influenced by the presence of a conspecific already feeding under the light, demonstrating that social facilitation occurs in free-ranging cane toads. Trials in which a toad was already feeding under the light attracted another toad sooner (mean = 37 min, *n* = 20 toads) than did trials without an already-feeding toad (mean = 58 min, *n* = 19 toads: *F*_1,37_ = 5.55, *P* = 0.024; see Figure 2a). There were no significant differences in average body length (asocial toads = 92.7 mm SE 2.1; social toads = 96.9 mm SE 2.4; *F*_1,94_ = 1.627, *P*>0.2), body mass (asocial toads = 105.17 g, SE 7.4; social toads = 109.76 g, SE 8.1: *F*_1,94_ = 0.1735, *P>*0.67) or body condition (*F*_1,94_ = 0.31, *P* = 0.57) between asocial and social toads.

### Personality Trials

Based on their behaviour during our field experiments, we predicted that asocial toads (i.e., those that approached the empty mats) were ‘bolder’ than social toads (those that approached already-occupied mats). When tested in the laboratory the following night, asocial toads took less time to emerge from a refuge and begin exploring a new environment (mean = 6.7 min) than did social toads (mean = 22.4 min; ANOVA: *F*_1,61_ = 14.85, *P*<0.0003; see Figure 2b). This behaviour was consistent between and within individuals when tested for a second time (repeatability = 0.73, SE = 0.08).

These results support our hypothesis that a toad’s personality influences its reliance on social cues when choosing foraging sites in the field; asocial toads tended to be bold whereas social toads were shy. The repeated-measures analysis showed that asocial toads, left the refuge sooner than did social conspecifics, regardless of whether or not they had been given ad libitum food immediately beforehand (personality effect: *F*_1,32_ = 6.44, *P*<0.02; feeding effect: *F*_1,32_ = 0.56, *P*>0.48). The interaction term was not significant (i.e., feeding did not delay the time to exit from the refuge for either bold or shy toads; personality*treatment effect: *F*_1,32_ = 3.97, *P* = 0.054). Repeatability of the time to exit the shelter was 0.77 (SE = 0.07), when feeding treatment was included as a factor in the model [34].

### Competition Trials

**Figure 3:**
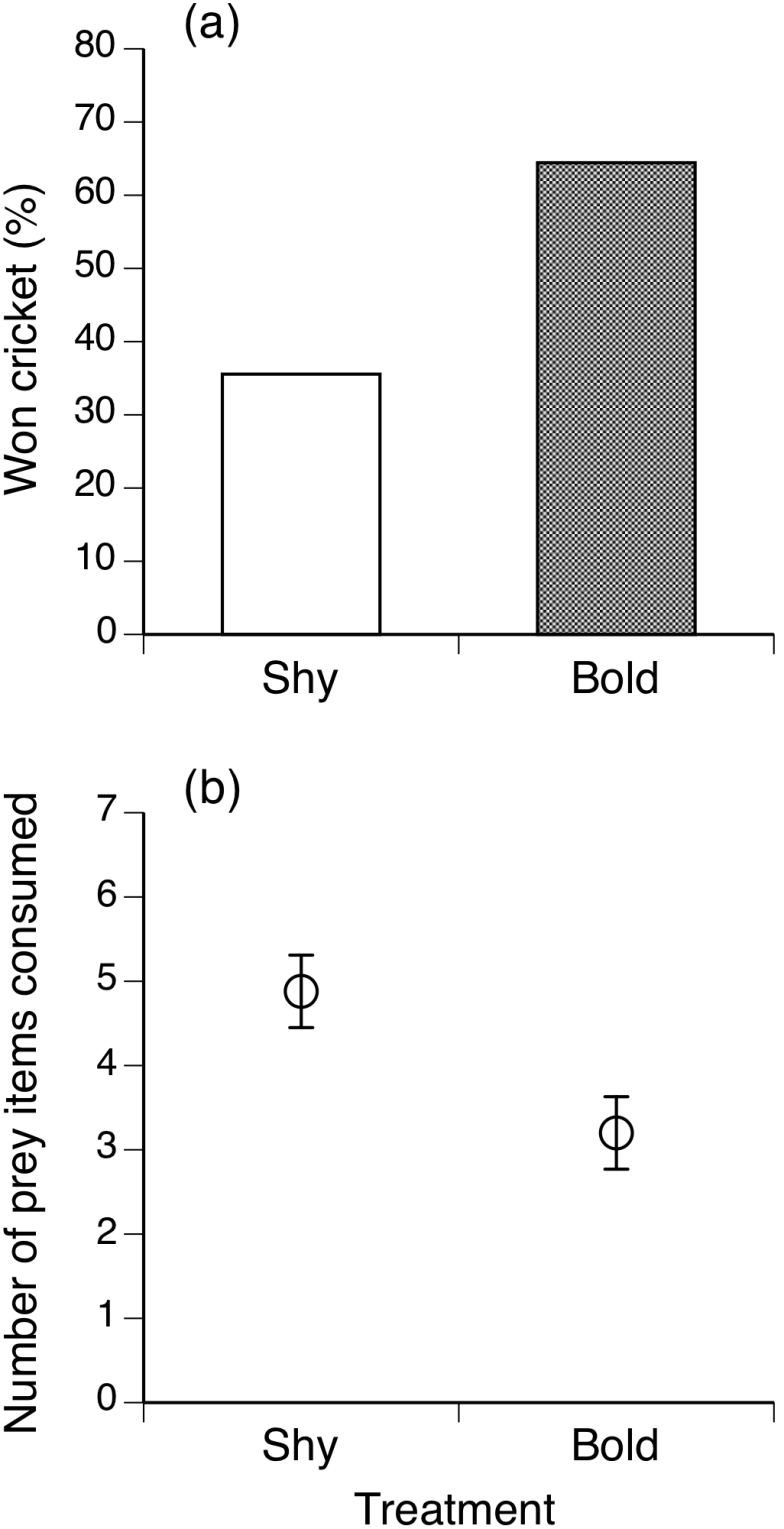
Foraging differences between ‘asocial-bold’ and ‘social-shy’ cane toads. Comparisons between ‘asocial-bold’ and ‘social-shy’ toads (classified based upon their responses to social cues in prior field trials) in terms of their foraging behaviour in standardized contests in the laboratory. When only a single cricket was available, the bolder individual was more likely to obtain that item (upper panel), but when multiple prey items (crickets) were available, shy toads ate more than bold toads (lower panel). The graphs show mean values and associated standard errors.

During the competition trials with a single cricket ‘asocial-bold’ toads (i.e., those that had approached unoccupied feeding stations in the field) were more accurate feeders (i.e., required fewer tongue-flicks to capture a cricket) in comparison to ‘social-shy’ conspecifics (*F*_1,57_ = 5.76, *P* = 0.01) and won the prey in 64% of cases (against a null of 50%: likelihood ratio χ^2^ = 7.61, 1 df, *P* = 0.005; see Figure 3a). However, the mean time taken to catch the cricket did not differ significantly between toads of the two personality types (*F*_1,43_ = 0.40, *P* = 0.52). This result was reversed if multiple crickets were provided, with social-shy toads gaining more prey items than their asocial-bolder competitors (*F*_1,48_ = 6.74, *P = *0.01; see Figure 3b).

## Discussion

We identified variation in foraging decisions made by free-ranging cane toads, based upon their sociality level as evaluated by their willingness to approach a novel unoccupied foraging site (versus a foraging site already occupied by a conspecific). We predicted that this variation in foraging decisions would be related to an individual’s position along the bold-shy continuum); that is, a behavioural syndrome between sociality and personality. If so, laboratory experiments should reveal different personality type in the toads that we had identified as ‘asocial’ versus ‘social’, based on the field experiment. Our results supported this prediction: a toad’s responses to social cues in the field predicted its subsequent behaviour in laboratory tests. Thus, our study population of toads appears to contain individuals with different personalities; and these traits influence ecologically significant behaviours such as the selection of foraging sites. Toads that were reluctant to approach a novel foraging area without the social stimulus of the presence of another toad (social), also were more hesitant to leave their shelter in laboratory arena trials, a common pattern in shy individuals [31], [32], [33]. In contrast, toads that did not need the social stimulus were bolder, soon leaving their shelter in the laboratory trials. These correlated behaviours support the existence of behavioural syndromes that involve both sociality-shyness and asociality-boldness among invasive cane toads from the population studied. Similar correlated behaviours have been detected in other species of fish and birds, where shyness is associated with the propensity to use social cues, and boldness with a higher exploratory behaviour that allows individuals to behave independently of social stimuli [17], [36], [37], [38], [39].

Nevertheless, behavioural traits such as willingness to approach a specific type of feeding site, or latency to exit from a shelter, undoubtedly are influenced by proximate factors as well as underlying ‘personality’ differences among individuals. Most obviously, hunger can influence an individual’s foraging decisions. For example, the hungrier an individual is, the greater the predation risk it may be willing to undergo in order to forage. A less hungry individual might choose to forage with a large group, where the increase in feeding competition is compensated for by a lower predation risk [40], [41]. Although it is logistically difficult to tease apart the influence of such proximate factors, our laboratory trials suggest that the behavioural differences between our ‘bold’ and ‘shy’ toads were not a simple result of differential hunger status: manipulating hunger level did not alter a toad’s propensity to leave its shelter. Toads that had been fasted for 48 hours did not leave their shelters faster (as would be expected if hunger level increased risk-taking), nor did toads that were fed until satiation take longer to emerge from their shelters. These results suggest that the variation in individual behaviours between ‘shy’ and ‘bold’ is not a simple consequence of differences in hunger-state levels. The pattern of emergence from the refuge was consistent with the differential reliance upon social cues as determined by our field trials (i.e., bold-asocial toads exited sooner than did shy-social toads). In addition, mean body condition did not differ between the two groups, suggesting that individual nutritional variation does not strongly affect either foraging site selection or the time taken to emerge from a shelter.

Both individual personality and level of sociality may influence the decisions that an organism takes, and thus also affect its foraging strategy. For example, when testing the effect of personality type and its role on the producer-scrounger game in barnacle geese (*Branta leucopsis*), shy individuals tended to join bold individuals in a foraging situation, showing that personality affects scrounging behaviour. In this case, bold geese led while shy geese, by using social information, followed [42]. Bold behaviour allows individuals to be more exploratory and to take higher risks to locate and exploit potential foraging sites. In this sense, bold cane toads play a role as finders (producers in the producer-scrounge game) by detecting rich foraging patches while shy toads behave as “followers” (scroungers) and benefit from social information (not only about prey availability, but also about an absence of predators) provided by the bold toads. In our own study, shy toads only approached sites that provided potential direct information not only about prey availability, but also about an absence of predators; a common pattern in other organisms [43], [44].

Although bold cane toads were able to detect and approach new unoccupied foraging patches, they took longer to identify them. This delay can be interpreted as a ‘disadvantage’, but might be compensated-for by an increased food intake due to the lack of competitors in the patch. For example, in *Poecilia reticulata,* bold females avoided areas where other conspecifics were feeding, probably as an interpretation of patch depletion [17]. Shy toads, on the other hand, by relying on the information received from bolder conspecifics, were able to reduce the time needed to detect and approach a foraging patch. In this way, the reduced food intake caused by higher competition (due to the presence of conspecifics) might be reduced via a longer time spent feeding (because of a reduced time taken to locate a foraging patch and survey for predators). This pattern has also been observed in guppies (*Poecilia reticulata*), where shy individuals tended to follow bold individuals to sources of prey, thereby reducing the average time to approach prey patches and increasing their food intake in comparison to conspecifics from groups composed entirely of shy individuals [36].

The fitness consequences of these divergent behavioural tactics will depend upon details of the local ecological context. For example, boldness may reduce an individual’s fitness if predators are abundant, but increase its fitness if predators are absent [45]. Our feeding competition trials suggest that bolder animals may be better competitors when prey abundance is low, but not when prey abundance is high. The mechanistic explanation for this difference remains to be explored, but our observations suggest that activity levels may be important. The ‘bold’ toads tended to be more active than their ‘shy’ conspecifics. They thus emerged from shelters sooner, and seized the first prey item sooner – but before long, began to move about the container apparently attempting to escape. Thus, ‘bold’ toads appeared to rapidly lose interest in feeding, and shift their attention to escaping. In contrast, the more sedentary ‘shy’ toads emerged later, but then settled into feeding without attempting to escape. This pattern may reflect the less active behaviour of shy toads, which allowed them (under our experimental settings) to focus on stimuli from the prey and as a consequence consume more prey.

These results accord with studies on rodents, where coping styles have been defined as proactive (analogous to boldness in studies with fish and birds) and reactive (analogous to shyness). In general, proactive individuals act based on prior experience (and so, tend to be quick but imprecise) whereas reactive individuals rely more on environmental information (leading to slower but more accurate responses to existing conditions [46]). For example, when applying an “anxiety test” to mice, aggressive males reacted with active swimming and climbing whereas non-aggressive males mainly expressed floating behaviour [47]. In hamsters, aggressive individuals were more prone to press a lever for a fast but small reward in comparison to less aggressive individuals, which obtained a larger reward by delaying their response [48]. The difference in behaviour among personalities during the competition trials in our study might also reflect the way in which shy and bold toads cope with stress. Bolder toads were more active and thus presented a more proactive coping style, whereas shyer toads remained almost immobile for longer periods analyzing the environment (a reactive coping style). Differences in behaviour might also reflect underlying differences in neurobiology and/or neuroendocrinology [49]. In other words, behaviours are correlated because they share neurobiological, neuroendocrine and/or genetic mechanisms. These mechanisms allow the behavioural flexibility that enables organisms to cope with environmental changes [46], [49]. High behavioural flexibility then, may enhance the ability to adapt to a changing environment, suggesting that successful invasive species will exhibit this condition.

Cane toads arrived at our study area only a few years before we conducted these trials, and it is interesting to consider the potential effects of behavioural syndromes on colonization processes. The mathematical models of Fogarty suggest that intraspecific variation in behavioural traits may facilitate invasion success [12]. That is, introduced species may spread more quickly when the population comprises a mixture of individuals with different personalities rather than being behaviourally monomorphic. High densities would promote the movement of asocial individuals to unoccupied areas, later colonized by social individuals [12]. Behavioural studies have confirmed that shy organisms may follow bold ones into new areas (e.g., in foraging fish *Poecilia reticulata)*, or bolder individuals may play a leading role in moving groups [44], [50]. The high densities attained in invasive populations might facilitate dynamics of this kind [20]. In cane toads (at least our studied population), most of these situations are present. The population includes individuals with a range of personality types that influence behavioural decisions taken in the presence of conspecifics, a pattern that is detectable due to the high densities attained in the area. Perhaps the most surprising and non-intuitive result of our study is to suggest a degree of cryptic sociality in an animal (the cane toad) that we would not have expected to show such disparities in individual responses to social cues. In turn, that result suggests that it will be worth conducting simple experiments to look for similar complexity in other invasive and non-invasive species of ectothermic vertebrates.
